# Boosting Immunogenicity of a Recombinant *Mycobacterium smegmatis* Strain via Zinc-Dependent Ribosomal Proteins

**DOI:** 10.3390/biomedicines12071571

**Published:** 2024-07-15

**Authors:** Shivani Singh, David Kanzin, Sarah Chavez, Noemi Alejandra Saavedra-Avila, Tony W. Ng, Regy Lukose, Oren Mayer, John Kim, Bing Chen, Mei Chen, Steven A. Porcelli, William R. Jacobs, Sangeeta Tiwari

**Affiliations:** 1 Department of Microbiology & Immunology, Albert Einstein College of Medicine, New York, NY 10461, USA; 2 Department of Medicine, Albert Einstein College of Medicine, New York, NY 10461, USA; 3 Department of Biological Sciences and Border Biomedical Research Center, University of Texas, El Paso, TX 79968, USA

**Keywords:** tuberculosis, vaccine, zinc, rpmb2, BCG

## Abstract

Tuberculosis (TB) continues to be a major global health burden and kills over a million people annually. New immunization strategies are required for the development of an efficacious TB vaccine that can potentially induce sterilizing immunity. In this study, we first confirmed that a live vaccine strain of *Mycobacterium smegmatis*, previously designated as IKEPLUS, conferred a higher survival benefit than the Bacillus Calmette-Guerin (BCG) in a murine model of intravenous *Mycobacterium tuberculosis* (Mtb) infection. We have shown that there was a significant increase in the expression of the Rv0282 gene, which is encoded in the *esx-3* locus, which played an important role in iron uptake when IKEPLUS was grown in both low zinc and iron-containing Sauton medium. We then confirmed using in vitro assays of biofilm formation that zinc plays a vital role in the growth and formation of *M. smegmatis* biofilms. IKEPLUS grown in low zinc media led to the better protection of mice after intravenous challenge with a very high dosage of Mtb. We also showed that various variants of IKEPLUS induced apoptotic cell-death of infected macrophages at a higher rate than wild-type *M. smegmatis*. We next attempted to determine if zinc containing ribosomal proteins such as rpmb2 could contribute to protective efficacy against Mtb infection. Since BCG has an established role in anti-mycobacterial efficacy, we boosted BCG vaccinated mice with rmpb2, but this did not lead to an increment in the protection mediated by BCG.

## 1. Introduction

Tuberculosis (TB) continues to be a major global health burden and kills over a million people annually [1]. Despite its questionable efficacy to prevent adult pulmonary TB, the attenuated *Mycobacterium bovis* strain Bacillus Calmette-Guerin (BCG) is the only licensed TB vaccine and continues to be widely used [2]. By virtue of being a live vaccine, BCG has restricted use in subjects most vulnerable to TB such as individuals living with HIV, and the generation of safer and more efficacious vaccines is therefore paramount. This requires a better understanding of the mechanisms that *Mycobacterium tuberculosis* (Mtb) deploys to evade the host immune response. One such mechanism is known to involve the specialized mycobacterial type VII secretion system known as ESX-1 [3,4]. An intact *esx-3* locus has been shown to be essential for the growth and survival of Mtb [5,6] but how exactly the *esx-3* locus contributes to mycobactericidal evasion is unknown. The expression of the *esx-3* genes is regulated by zinc, suggesting that zinc may have a key role to play in Mtb virulence [7,8]. In line with others who have developed recombinant strains [9,10], we had previously constructed a vaccine strain by introducing the Mtb *esx-3* genes into a *Mycobacterium smegmatis* (msmeg) Δesx-3 mutant strain [11]. We observed a loss of the ability to evade bactericidal host innate immunity for this msmeg strain, which we therefore designated by the acronym IKE to signify ‘immune killing evasion’, and since the insertion of the Mtb *esx-3* genes into IKE produced a recombinant strain that did not reverse the susceptibility of the bacteria to innate immune killing, we called it IKEPLUS [11]. Compared to BCG, the immunization of C57BL/6 mice with IKEPLUS led to marked reductions in lung, spleen, and liver bacterial burdens as well as prolonged survival after Mtb challenge [11]. Inoculation with IKEPLUS induced a more robust Th1 cytokine milieu with a rapid IFN-γ and IL-12 response and increased phagosome maturation during macrophage infection.

We then sought to determine the mechanism of bacterial killing in IKEPLUS-immunized mice as a crucial next step. Since protective immunity induced by IKEPLUS was dependent on antigen-specific CD4^+^ T-cell responses, we hypothesized that the specificity of this response would be a critical feature of its protective phenotype. Using IFN-γ enzyme-linked immunosorbent spot (ELISPOT) assays on splenocytes from IKEPLUS-vaccinated mice, we identified an immunogenic peptide within the mycobacterial ribosomal structural protein RplJ [12]. Previous studies have shown the zinc dependence of various ribosomal proteins [13] as well as the induction of ribosomal hibernation by the depletion of zinc in mycobacteria [14]. In a complementary approach, we generated major histocompatibility complex (MHC) class II-restricted T-cell hybridomas from IKEPLUS-vaccinated mice and demonstrated that the CD4^+^ T-cell response recognized several components of the mycobacterial ribosome and was localized in the lungs after Mtb challenge. We then extended our investigations by broadly screening a collection of more than 20 structural proteins of the Mtb ribosome for their ability to be targeted by CD4^+^ T-cells in IKEPLUS immunized mice and showed that the mycobacterial ribosome was highly immunogenic [15]. The induction of strong multifunctional Th1 CD4^+^ T-cell responses and reduction of lung pathology and bacterial burdens were noted in RplJ-boosted mice after Mtb challenge [15]. With the above background, we tested the hypothesis that vaccination strategies directed at priming CD4^+^ T-cells against zinc-dependent ribosomal proteins such as rpmb2 could be considered an approach to enhance vaccine efficacy against Mtb.

## 2. Methods

### 2.1. Bacterial Strains, Plasmids, Phages and Media

All msmeg strains used in this study were derived from the laboratory parent strain mc^2^155. Descriptions of all strains and plasmids used in this study are provided in Supplementary Tables S1–S3. Msmeg strains were cultured in 7H9 liquid medium (Difco, Becton-Dickinson, Franklin Lakes, NJ, USA) containing 0.5% glycerol, 0.2% dextrose, and 0.05% Tween-80 for mutant construction. The strain was cultured in regular Sauton medium (RS) containing 0.05% Tween-80 for animal infections [11]. Sauton medium used for the growth of msmeg strains is a liquid medium consisting of 60 mL/L of glycerol, 0.5 g/L of KH_2_PO_4_, 2.0 g/L of citric acid monohydrate, 4 g/L of asparagine, 0.5 gm/L of magnesium sulfate, 1 mg/L of zinc sulfate, and 50 mg/L of ferric ammonium citrate. The pH of the solution was adjusted to 7.0–7.2 with the addition of 10 N sodium hydroxide. For low zinc and low iron-containing Sauton media (liquid medium), the final concentration of zinc was 100 μg/L, and the final concentration of iron was 0.5 mg/L. The BCG Danish SSI strain used for immunizations was from AERAS (a non-profit organization dedicated to the development of effective TB vaccines and biologics), and the Mtb strain was the H37Rv laboratory strain derivative obtained from the Trudeau Institute. Mtb and BCG were cultured using Middlebrook 7H9 liquid medium (Difco, Becton-Dickinson, Franklin Lakes, NJ, USA) containing 10% OADC (oleic acid/albumin/dextrose/catalase), 0.5% glycerol and 0.05% Tween-80. Media were supplemented with ‘as required’ antibiotics hygromycin (50 μg/mL) or apramycin (20 μg/mL). Colony counts were obtained by plating on 7H10 agar plates containing 10% OADC, 0.5% glycerol, and 0.05% Tween-80.

### 2.2. Generation of IKEPLUS

Specialized transducing phage (phAE543), as described previously [11,16], was used to generate MsΔ*esx-3* (Ms0615-Ms0626) or mc^2^6462. Transductions were plated on 7H10 plates containing 150 µg/mL hygromycin. Hygromycin-resistant clones were screened for deletion by polymerase chain reaction (PCR) and further confirmed by whole genome sequencing. Antibiotic marker was removed using phage pHAE280 to generate the unmarked strain (mc^2^6463 or IKE) followed by transformation of this strain with cosmid pYUB2098 (Rv0278-Rv303)::*kan^R^* to generate mc^2^6456. As this strain contains an integrase that can lead to instability of the cosmid pYUB2098(Rv0278-Rv303)::*kan^R^*, mc^2^6456 was transformed with a plasmid bearing resolvase (pYUB2099) and named mc^2^7159 (Stable IKEPLUS). Furthermore, to transform this strain with an immunoreporter without incorporating an antibiotic marker, we deleted *leuCD* gene in mc^2^7159 to generate mc^2^7170 and unmarked it to make mc^2^7173 using phAE280. Finally, mc^2^7173 was transformed with immunoreporter PBRL635 containing immunogenic epitopes of ESAT-6-SINFIKEL-OVAII on a plasmid with *leuCD* genes as a selection marker (instead of antibiotic) and called mc^2^7257 (SIPΔ*leuCD*::pBRL635 *leuCD*). In our murine survival experiments, we compared this IKEPLUS strain with the previously generated IKEPLUS strain mc^2^5009 [11].

### 2.3. Q-PCR

In to determine if the expression of ESX-3 in IKEPLUS changes in response to low iron and low zinc conditions, we performed Q-PCR. The mc^2^5009 and mc^2^7182 strains were cultured in regular Sauton (RS), low iron Sauton (LFeS), and low zinc Sauton (LZnS) media. Cultures were pelleted and suspended in ‘RNA protect’ for storage until further processing. To isolate RNA, samples were centrifuged, and the pellets were suspended in 1 mL trizol, followed by bead-beating (3 times/30 s). Supernatants were collected and an equal volume of ethanol was added. Further purification was performed as per manufacturers’ protocol (Zymo-spin RNA purification kit, Zymo Research Corporation, Irvine, CA, USA). Synthesis of cDNA was performed as per protocol in the Superscript III Reverse Transcriptase kit (Invitrogen#18989-093, Life Tech., Carlsbad, CA, USA). This was followed by Q-PCR using Sybr green mix (Promega, Madison, WI, USA). Forty cycles were performed, and relative expression of Rv0282 was determined in RS, LFeS, and LZnS media after normalizing with 16S RNA.

### 2.4. Murine Infections

C57BL/6 mice (6–8 weeks old, both male and female) were obtained from the Jackson Laboratory, USA (an independent and non-profit biomedical mammalian research institution). All msmeg strains used for immunization of mice were cultured either in RS or LZnS media. BCG Danish SSI was grown in 7H9 media. Before inoculation, bacteria were washed twice with PBS plus 0.05% Tween 80. Subsequently, bacteria were sonicated twice for 10 s each time and then diluted to achieve the desired densities. Different groups of mice were immunized intravenously (i.v.) with BCG/IKEPLUS strains grown in either RS or LZnS media, or sham immunized with i.v. PBS. After 7–8 weeks of immunization, mice were challenged with high dose of Mtb strain H37Rv (5.5 × 10^7^ or 10^8^ CFU/mouse) via the i.v. route. The mice were observed without any further intervention for survival studies. Statistical analysis was done using log-rank test. The animal protocol was approved by the University of Texas at El Paso and Albert Einstein College of Medicine Institutional Animal Care and Use Committee (IACUC) protocols, as per NIH principles for animal usage.

### 2.5. Boosting BCG-Primed C57BL/6 Mice with rpmb2 Ribosomal Protein

C57BL/6 mice were vaccinated with 5 × 10^6^ CFUs of BCG Danish via the subcutaneous (SQ) route. After intervals of 6 weeks and 8 weeks, the BCG primed mice were boosted via the SQ route with 25 μg of rpmb2 mixed with the adjuvant CpG ODN type B. CpG oligodeoxynucleotides (or CpG ODN) are short single-stranded synthetic DNA molecules that contain a cytosine triphosphate deoxynucleotide (C) followed by a guanine triphosphate deoxynucleotide (G). They are a new class of Th1-type immune stimulants that stimulate the TLR9 receptor and in doing so have proven to be a highly efficacious adjuvant for mucosal vaccines against infectious diseases. The prime-boosted mice were aerosol infected with H37Rv at 12 weeks after BCG prime, and the mice were euthanized for organ retrieval (lung, spleen, and thoracic lymph nodes) and CFU enumeration at 10 weeks after H37Rv challenge.

### 2.6. Polycaspase Activation Assays

Bone marrow macrophages (BMM) were isolated and differentiated in cultures supplemented with M-CSF as described previously [16]. Peritoneal macrophages (PM) were prepared by injecting 2 mL of 3% Brewer Thioglycolate into the peritoneal cavity of mice. Four days after inoculation, mice were euthanized, and peritoneal macrophages were extracted from peritoneal cavity by injecting 5 ml of PBS and withdrawing the same volume of fluid from the peritoneal cavity. The cells were centrifuged, washed, and suspended in complete DMEM medium for plating. After 24 h, cells were washed to remove suspended cells, and adhered macrophages were cultured. After 6–7 days, macrophages were trypsinized, counted, and seeded for infection assays. All the *M. smegmatis* strains were grown in LZnS media. At 24 h after seeding, macrophages were infected for 3 h with the respective strains at a multiplicity of infection (MOI) of 1:10. Cells were washed and stained with FLICA 660 PolyCaspase kit (ImmunoChemistry Technologies, Davis, CA, USA, Catalog#9120) 24 or 48 h post-infection to measure apoptosis as a surrogate measure of active caspase enzymes. For flow cytometry, cells were stained as per manufacturer’s instructions and samples were analyzed on the FACS Aria flow cytometer.

### 2.7. Biofilm Assay

Sauton Media was prepared as mentioned above. Zinc and iron supplements were added as indicated. Log phase cultures of the desired strains were pelleted by centrifugation (300 G for 10 min at RT). This was followed by resuspending the cells in 10 mL Sauton media and re-suspending the centrifuged pellet with Sauton media to the original volume of the culture. Biofilm growth was performed on a 12-well polystyrene plate. Wells were inoculated with cells at a ratio of 1:50 cells to the total volume of the media in the well. Plates were wrapped in the foil and incubated at 37 °C, 5% CO_2,_ and checked visually on a weekly basis for biofilm growth.

### 2.8. Expression of Mtb Ribosomal Protein rpmb2 in E. coli Plasmids

Production of recombinant Mtb ribosomal protein rpmb2 was undertaken in collaboration with the protein core using *E. coli* harboring plasmids that encoded these ribosomal proteins. Auto-induction was done using Isopropyl β-D-1-thiogalactopyranoside (IPTG) as the auto-induction reagent. Purification was undertaken using nickel affinity chromatography, size-exclusion chromatography, and ion exchange chromatography, followed by overnight dialysis against cell-culture grade PBS. Next, endotoxin was reduced by overnight incubation with endotoxin removal resin. Finally, protein concentration was measured by UV absorbance at 280 nm and endotoxin load was determined using a Limulus amebocyte lysate assay.

### 2.9. Generation of rpmb2 Specific T-Cell Hybridoma and IL-2 ELISA

C57BL/6 mice were immunized with 10^7^ CFU of IKEPLUS via the intravenous route. After intervals of 4 weeks and 6 weeks, these mice were injected with 25 μg rpmb2 mixed with 20 μg of CPG ODN via the intraperitoneal route. Two weeks after the last boost, the mice were sacrificed, and spleen and mesenteric lymph nodes were harvested. Using the Miltenyi Biotec kit (catalog #130-104-454, BD Biosciences, Franklin Lakes, NJ, USA), which used CD4^+^ T-cell biotin-antibody cocktail and anti-biotin microbeads, CD4^+^ T-cells were isolated from the spleen and mesenteric lymph nodes and incubated with rpmb2 and bone marrow-derived dendritic cells for 3 days. Next, these CD4^+^ T-cells were fused with T-cell receptor-negative BW5147 thymoma cells as per previously published guidelines [17,18]. The fused cells (hybridomas) were expanded and were then subjected to a limiting dilution. The primary hybridomas were pre-screened for TCR-β expression using flow cytometry. BCG-infected bone-marrow-derived dendritic cells were incubated overnight with T-cell hybridomas specific for rpmb2 or Ag85B. Supernatants were harvested and the levels of IL-2 were quantitated by a capture ELISA as previously described [19]. Absorbance values were determined at 450 nm on a Wallace 1420 Victor2 microplate reader (PerkinElmer, Waltham, MA, USA) and values for IL-2 were determined by using a standard curve generated by using known concentrations of purified recombinant murine IL-2 (BD Biosciences, Franklin Lakes, NJ, USA).

## 3. Results

### 3.1. Immunization with IKEPLUS Strains Protect Mice against Challenge with High Dose Mtb

**Figure 1:**
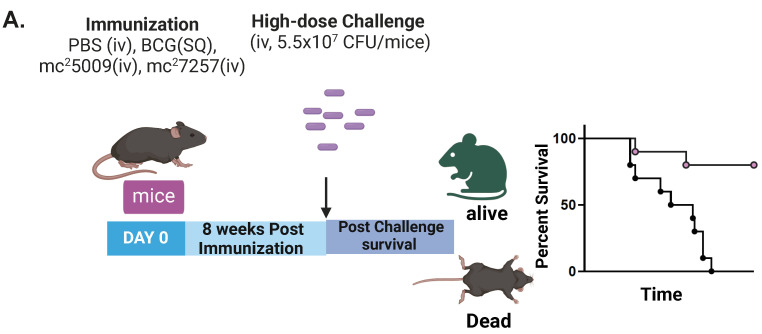

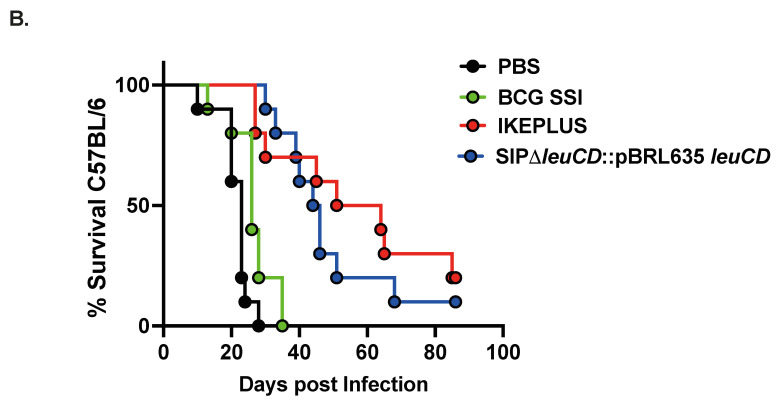
Immunization with IKEPLUS strains protects mice against challenge with high-dose Mtb. (**A**) Experimental design for immunization and challenge for survival studies. 6–8 weeks old C57BL/6 mice (n = 10) were sham immunized with i.v. PBS, SQ BCG (10^7^ CFU/mouse), and SQ IKEPLUS strains SIPΔ*leuC*D::pBRL635 *leuCD* (mc^2^7257) or IKEPLUS (mc^2^5009) with approximately 5 × 10^8^ CFU/mouse grown in the RS media. After 8-weeks of immunization, mice were challenged with a high dose of i.v. Mtb strain H37Rv as shown by arrow. (**B**) Differences in the survival curves were significant for PBS versus SIPΔ*leuCD*::pBRL635 *leuCD* strain (*p* = 0.0001, log-rank test) as well as PBS versus IKEPLUS strain (*p* = 0.0001, log-rank test). In addition, there was a survival benefit when mice were immunized with SQ BCG (*p* = 0.01).

Mice (6–8 week old C57BL/6) (n = 10) were immunized with i.v. PBS, SQ BCG (10^7^ CFU/mouse), and SQ IKEPLUS strains SIPΔ*leuCD*::pBRL635 *leuCD* (mc^2^7257) or IKEPLUS (mc^2^5009) with approximately 5 × 10^8^ CFU/mouse grown in the RS media as per the strategy illustrated in Figure 1A. After 8 weeks of immunization, the mice were challenged with a high dose of i.v. Mtb strain H37Rv (5.5 × 10^7^ CFU/mouse). The mice were observed for survival studies as shown in Figure 1B. Differences in the survival curves were significant for PBS versus SIPΔ*leuCD*::pBRL635 *leuCD* strain (*p* = 0.0001, log-rank test) as well as PBS versus IKEPLUS strain (*p* = 0.0001, log-rank test). The difference observed between the protective efficacies of the two IKEPLUS strains was insignificant and could be attributed to the difference in the CFU delivery to the mouse lungs (target dose was 5 × 10^8^ /mouse but CFU delivered was 7 × 10^8^ for mc^2^5009 strain). In addition, there was a survival benefit when mice were immunized with SQ BCG, in keeping with previous murine studies on BCG (*p* = 0.01).

### 3.2. LZnS Medium Enhances IKEPLUS Mediated Protection against High Dose of i.v. Mtb Challenge

**Figure 2:**
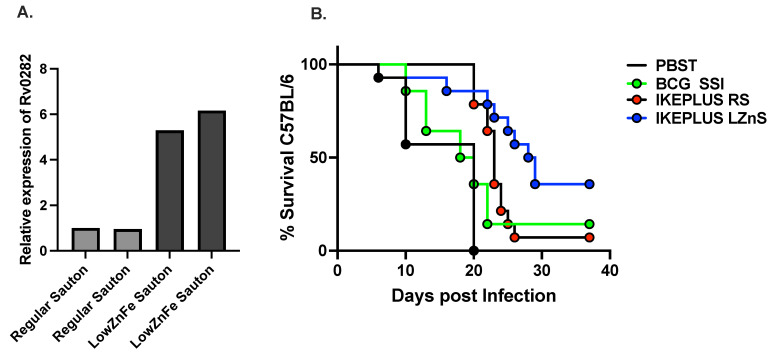
LZnS medium enhances IKEPLUS-mediated protection against high doses of i.v. Mtb challenge. (**A**) A five-fold increase in the expression of the Rv0282 when IKEPLUS was grown in low zinc Sauton (LZnS) and low iron Sauton (LFeS) media compared to RS medium was noted. (**B**) C57BL/6 mice were immunized with i.v. PBS Tween (PBST), SQ BCG (10^7^ CFU/mouse), and the IKEPLUS strain mc^2^5009 (5 × 10^7^/mouse) grown in RS or LZnS media. Seven weeks after immunization, mice were challenged with high-dose H37Rv. IKEPLUS/mc^2^5009 grown in LZnS media led to better protection of mice compared to mice immunized with IKEPLUS/mc^2^5009 grown in RS (*p* = 0.03). Differences in the survival curves were significant for both mc^2^5009/IKEPLUS grown in RS (*p* = 0.001, log-rank test) and LZnS (*p* = 0.001, log-rank test) when compared to IKEPLUS grown in PBS.

Here, we tested whether modulating the level of zinc and iron in Sauton growth medium can regulate the expression of the Mtb ESX-3 gene expressed in *M. smegmatis*. We found that there was a five-fold increase in the expression of the Rv0282 (the first gene in the ESX-3 operon) when IKEPLUS was grown in LZnS and LFeS compared to RS medium (Figure 2A). Biofilm assays showed that wild-type *M. smegmatis* mc^2^155 grown in 7H9 media formed thin biofilms, but the same strain grown in Sauton medium formed thick biofilms (Figure S1A). Additionally, when the old Sauton medium was supplemented with zinc and iron, *M. smegmatis* growth was augmented Figure S1B. Next, we investigated the role of zinc in the growth of wild-type *M. smegmatis*, IKEPLUS, and IKE. Zinc concentrations were titrated to support the growth of *M. smegmatis.* We found that zinc plays a vital role in the growth and formation of *M. smegmatis* biofilms, and 100 μug/L of zinc sufficiently supported the growth of *M. smegmatis* (Figure S1B). Therefore, we choose low zinc (100 μg/L) Sauton media in further infection studies. C57BL/6 mice (n = 14) were immunized with i.v. PBS, SQ BCG (10^7^ CFU/mouse), and the IKEPLUS strain mc^2^5009 (5 × 10^7^/mouse) grown in RS or LZnS media. Seven weeks after immunization, the mice were challenged with a high dose of i.v. Mtb strain H37Rv (1 × 10^8^ CFU/mouse). Mice were observed for survival studies. IKEPLUS/mc^2^5009 grown in LZnS media led to better protection of mice after i.v. challenge with a very high dosage of Mtb (1 × 10^8^/mouse) compared to mice immunized with IKEPLUS/mc^2^5009 grown in RS (Figure 2B, *p* = 0.03). Differences in the survival curves were significant for both mc^2^5009/IKEPLUS grown in RS (*p* = 0.001, log-rank test) and LZnS (*p* = 0.001, log-rank test) when compared to IKEPLUS grown in PBS.

### 3.3. IKEPLUS Induces Apoptotic Cell-Death of Infected Cells Macrophages

**Figure 3:**
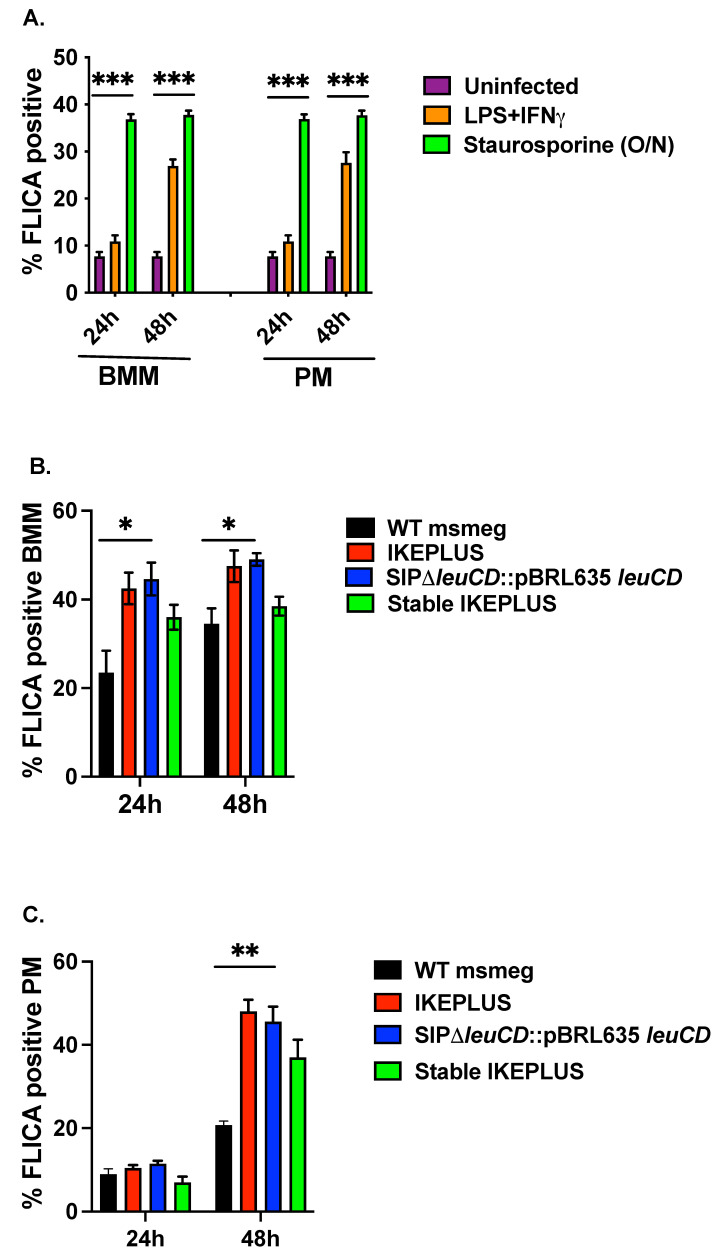
IKEPLUS induces apoptotic cell death of infected cells macrophages. (**A**) A three-to-four-fold increase in the apoptosis rate of bone marrow macrophages (BMM) or peritoneal macrophages (PM) treated with LPS (100 ng/mL) in combination with IFNγ (200 U/mL) or Staurosporine (1 μM overnight (O/N)); as compared to uninfected macrophages was noted. (**B**)There was an approximately 20% increase in the apoptosis observed in BMM treated with various variants of IKEPLUS compared to wild-type mc^2^155 within 24 hours. This increment decreased to 10% but was still significant at 48 h. (**C**) Infected PMs did not illustrate a significant difference in the activation of caspase/apoptosis at 24 h but a 30% increase in apoptosis was noted in the samples infected with IKEPLUS variants compared to wild-type mc^2^155 at 48 h. Mean ±SD is plotted (n = 3). (*** *p* < 0.001, ** *p* < 0.01 and * *p* < 0.05) by ANOVA.

Bone marrow macrophages (BMM) and peritoneal macrophages (PM) were infected with *M. smegmatis* strains grown in LZnS media at an MOI of 1:10 for 3 h. After 24 or 48 h post-infection, macrophages were washed and stained with fluorochrome-labeled inhibitors of caspases (FLICA, 660 Poly Caspase kit) that detect active caspase enzymes involved in apoptosis. Stained macrophages were read on FACSAria flow cytometer. For the negative control, we used uninfected (UI) macrophages, and for the positive control, macrophages were stimulated with either LPS (100 ng/mL) in combination with IFNγ (200 U/mL) or Staurosporine (1 μM overnight) for 24 or 48 h. We noted a three-to-four-fold increase in the apoptosis rate of BMM or PM macrophages treated with LPS (100 ng/mL) in combination with IFNγ (200 U/mL) or Staurosporine (1 μM overnight) as compared to uninfected macrophages (Figure 3A). There was an approximately 20% increase in the apoptosis observed in BMM treated with various variants of IKEPLUS compared to wild-type mc^2^155 within 24 hours (Figure 3B). This increment decreased to 10% but was still at 48 h (Figure 3B). Infected PMs did not illustrate a significant difference in the activation of caspase/apoptosis at 24 h, but a 30% increase in apoptosis was noted in the samples infected with IKEPLUS variants compared to wild-type mc^2^155 at 48 h (Figure 3C).

### 3.4. rpmb2 Does Not Augment BCG Mediated Protection against Mtb

**Figure 4:**
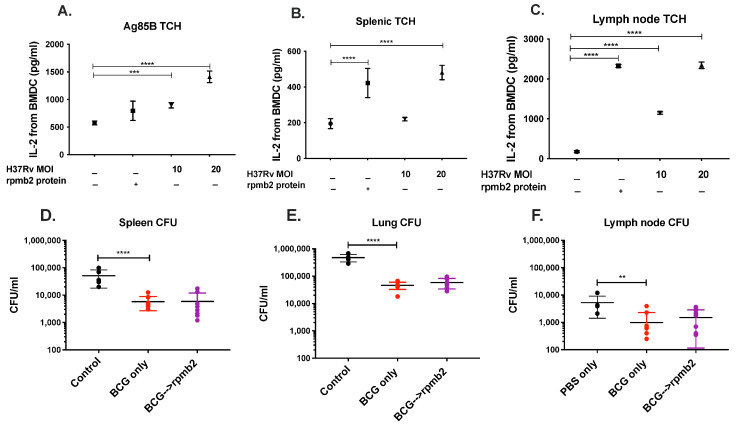
rpmb2 does not augment BCG-mediated protection against Mtb. (**A**–**C**) An incremental increase in the secretion of IL–2 upon incubation of rpmb2-specific and Ag85B-specific T-cell hybridomas with H37Rv-infected bone-marrow-derived dendritic cells (BMDC) was noted. IL–2 secretion increased with an increase in the MOI of H37Rv infection and was positive upon incubation with rpmb2 (**B**,**C**). Ag85B–specific T-cell hybridomas were used as a positive control and did not stimulate IL–2 secretion upon incubation with rpmb2 (**A**). (**D**–**F**) BCG–vaccinated mice were boosted with rmpb2, but this did not lead to an increment in the protection mediated by BCG, as illustrated by spleen, lung, and lymph node CFUs (**D**–**F**). ** for *p* < 0.001, *** for *p* < 0.0001, **** for *p* < 0.00001 by ANOVA.

Finally, we show an incremental increase in the secretion of IL-2 upon incubation of rpmb2 (a zinc-dependent ribosomal protein) specific T-cell hybridomas and Ag85B specific T-cell hybridomas with H37Rv-infected bone-marrow-derived dendritic cells (BMDC) (Figure 4A–C). IL-2 secretion increased with an increase in the MOI of H37Rv infection of BMDCs and was positive upon incubation with the ribosomal protein rpmb2. Ag85B-specific T-cell hybridomas were used as a positive control and did secrete IL-2 upon incubation with rpmb2. Having established that rpmb2-specific T-cell hybridomas are stimulated by H37Rv-infected bone-marrow-derived dendritic cells, we next attempted to determine if rpmb2 could contribute to protective efficacy against Mtb infection in a mouse model of TB infection. BCG has an established role in anti-mycobacterial efficacy. We boosted BCG-vaccinated mice with rmpb2, but this did not lead to an increment in the protection mediated by BCG, as illustrated by spleen, lung, and lymph node CFUs (Figure 4D–F).

## 4. Discussion

New immunization strategies are required for the development of an efficacious TB vaccine that can potentially induce sterilizing immunity and modulate the host immune system to eliminate virulent mycobacteria. Here, we hypothesized that vaccination strategies directed at priming CD4^+^ T-cells against zinc-dependent ribosomal proteins such as rpmb2 could be considered an approach to enhance vaccine efficacy against Mtb. Subdominant or cryptic antigens may have the potential to induce CD4+ T-cell responses that can result in bactericidal and protective immunity. First, we illustrated a survival difference when mice were immunized with IKEPLUS strains prior to Mtb infection. Since secretory antigens such as ESAT-6 and CFP-10 are secreted when Mtb is grown in Sauton media but not in 7H9 media [20], we grew all msmeg strains in Sauton. It has also been reported that some of the genes in the ESX-3 region are induced by iron [21] and that ESX-3 is under the regulation of Zur-FurB regulon [7]. We demonstrated a five-fold increase in the expression of the Rv0282 (first gene in the ESX-3 operon) when msmeg strains were cultured in either low iron or low zinc containing Sauton. Mice vaccinated with IKEPLUS/mc^2^5009 grown in low zinc Sauton media were better protected after Mtb challenge, as compared to msmeg strains grown in regular Sauton media. Finally, since rpmb2 is a zinc-dependent ribosomal protein, we constructed rpmb2-specific T-cell hybridomas from intravenous IKEPLUS vaccinated mice and tested their reactivity after incubating them with bone-marrow-derived dendritic cells that had been infected with H37Rv. Since BCG has an established protective efficacy against TB, we boosted BCG-vaccinated mice with subcutaneous rpmb2 but found that this did not have an incremental effect on the protective efficacy of BCG.

We first confirmed our previous finding [11] that various strains of IKEPLUS confer a higher survival benefit than BCG. This may in part be due to the fact that BCG has a better protective profile against Mtb infection via the pulmonary route rather than the i.v. route that was used in our study [22,23]. We have shown that there was a five-fold increase in the expression of the Rv0282 (the first gene in the ESX-3 operon) when IKEPLUS was grown in LZnS or LFeS medium compared to RS medium. This was confirmed on biofilm assays that showed that zinc plays a vital role in the growth and formation of *M. smegmatis* biofilms, and IKEPLUS/mc^2^5009 grown in LZnS media led to better protection of mice after i.v. challenge with a very high dosage of Mtb. We surmise that this may be due to different pools of antigens, such as ribosomal proteins, being induced/expressed under low zinc conditions [14]. We also showed that various variants of IKEPLUS induced apoptotic cell death of infected macrophages at a higher rate than wild-type mc^2^155. Upon incubation of rpmb2 (zinc-dependent ribosomal protein) specific T-cell hybridomas and Ag85B specific T-cell hybridomas with H37Rv-infected bone-marrow-derived dendritic cells, IL-2 secretion increased with an increase in the MOI of H37Rv infection of BMDC’s. Having established that rpmb2-specific T-cell hybridomas are stimulated by H37Rv-infected bone-marrow-derived dendritic cells, we next attempted to determine if rpmb2 could contribute to protective efficacy against Mtb infection in a mouse model of TB infection. Since BCG has an established role in anti-mycobacterial efficacy, we boosted BCG-vaccinated mice with rmpb2, but this did not lead to an increment in the protection mediated by BCG; however, in previous and ongoing studies, we have shown that boosting BCG-primed mice can lead to improved protective efficacy against Mtb [15].

Candidate vaccines against Mtb have largely focused on targeting immunodominant antigens such as the Ag85 complex, ESAT-6, and TB10.4, to name a few [24], but enhanced protection has been elusive, and safety issues have been frequent. Progress in this field has been hindered by a lack of knowledge about immune correlates of protection. The discovery that several known T-cell epitopes of Mtb are derived from hyper-conserved T-cell epitopes suggests that the recognition of these epitopes by the host may be beneficial to Mtb, thereby raising the possibility that there may be decoy antigens’ continued replication and transmission [25]. Such CD4^+^ T-cell epitopes, by virtue of not being directly involved in the host-pathogen coexistence, could represent more effective vaccine targets [12]. Using a synthetic peptide library consisting of 880 non-overlapping 15-mers representing predicted I-Ab-presented epitopes, our group had previously shown that the specificity of the CD4^+^ T-cells evoked by IKEPLUS and cross-reactive with Mtb were specific for structural proteins of the mycobacterial ribosome [12]. This CD4^+^ T-cell response was MHC class-II-restricted and dominated by the production of IFN-γ, suggesting the development of a Th1-like response. Taken together, these studies raise the possibility that immunization against mycobacterial ribosomal components primes the host immune response to recognize ribosomal epitopes when challenged with the virulent, ribosome-limited Mtb and that ribosome-specific CD4^+^ T-cells are recruited to and expand in the lungs of IKEPLUS primed and Mtb-challenged mice [12]. Ribosome vaccines are immunogenic, less toxic, and better defined than whole-cell vaccines and previous studies demonstrated the potential of ribosomal components as protective vaccines [26,27]. Components of the ribosome itself can act as adjuvants and stimulate both cell and humoral-mediated immunity. But the mechanism of recognition, processing, and presentation of ribosomes following infection or immunization remains unclear, as do the underpinnings of the development of ribosome-specific T-cell responses following infection [12].

One limitation of our study was that the bactericidal effects were most apparent when we administered IKEPLUS intravenously. This route is not the most feasible route for clinical trials, although recent animal studies on i.v. BCG have stimulated renewed interest in this route of vaccination. Further improvements will be needed to optimize the efficacy of IKEPLUS vaccination for implementation in humans, which may involve prime-boost regimens. Taken together, our findings suggest that the incorporation of cryptic antigens into vaccination strategies may be useful in promoting a protective CD4^+^ T-cell response against Mtb. Others have shown that cryptic antigens are able to prime a CD4^+^ T-cell response with greater TNF-α and IL-2 production and a higher proliferative capacity compared to immunodominant antigens [28]. An Mtb genome-wide screen to identify targets of the MHC class II-restricted CD4^+^ T-cell responses highlighted the sheer breadth of epitopes recognized during Mtb infection, emphasizing the potential importance of designing multiepitope vaccines [29].

## Data Availability

No new data were created or analyzed in this study. Data sharing is not applicable to this article.

## Supplementary Materials

The following supporting information can be downloaded at: https://www.mdpi.com/article/10.3390/biomedicines12071571/s1, Figure S1: Zinc and Iron affect the growth of *M. smegmatis* in Sauton medium; Table S1: List of strains used in the current study; Table S2: List of plasmids and phasmids used in the current study. Table S3: Primers used in the current study. References [11,30] are cited in the supplementary materials.
