# Supplementary material for: Boosting Immunogenicity of a Recombinant Mycobacterium smegmatis Strain via Zinc-Dependent Ribosomal Proteins
Source: Biomedicines. 2024 Jul 15;12(7):1571. doi: 10.3390/biomedicines12071571 (PMC11274837; doi:10.3390/biomedicines12071571)
Supplement: Supplementary file 1 [file biomedicines-12-01571-s001.zip › biomedicines-3028189-supplementary.pdf]

Table S1: List of strains used in the current study. Abbreviations used *hyg*^{R}: hygromycin cassette, *sacB*: counter selectable marker, k*an*^{R} kanamycin resistance.

Strain No	Strain name	Genotype	Description	Source/Reference
H37Rv	WT	Parent strain		Trudeau Institute
BCG SSI		Parent strain		AERAS
mc^{2}155	*ept-1*	Parent strain		(1)
mc^{2}6462		*ept-1* Δ(*Ms 0615-Ms0626*):: *hyg*^{R}, *suc*^{S}	Specialized transduction of mc^{2}155 with phAE543	Tiwari et al, (unpublished)
mc^{2}6463	IKE	*ept-1* Δ(*Ms 0615-Ms0626*)	Specialized transduction of mc^{2}6462 with phAE280	Tiwari et al, (unpublished)
mc^{2}6456	IKEPLUS_2	*ept-1* Δ(*Ms 0615-Ms0626*):: $attB_{L5}$ pYUB2098 *kan*^{R}	Transformation of mc^{2}6463 with pYUB2098 (Kan^R)	This study
mc^{2}7159	SIP	*ept-1* Δ(*Ms 0615-Ms0626*):: $attB_{L5}$ pYUB2098Δ*kan*^{R} Δintegrase	Transformation of mc^{2}6456 with pYUB2099 (containing delta gamma resolvase)	This study
mc^{2}7170		*ept-1* Δ(*Ms 0615-Ms0626*), $attB_{L5}$*::pYUB2098*Δ *kan*^{R}*,* Δ*leuCD (Ms2387-88), :: hyg*^{R}, *suc*^{S}	Specialized transduction of mc^{2}7159 with pHAE763	This study
mc^{2}7173		*ept-1* Δ(*Ms 0615-Ms0626*), $attB_{L5}$*::pYUB2098*Δ *kan*^{R}*,* Δ*leuCD (Ms2387-88*)	Specialized transduction of mc^{2}7173 with pHAE280	This study
mc^{2}7257	SIPΔ*leuCD:: PBRL635*	*ept-1* Δ(*Ms 0615-Ms0626*), $attB_{L5}$*::pYUB2098*Δ *kan*^{R}*,* Δ*leuCD + PBRL635 (leuCD)*	Transformation of mc^{2}7173 with pBRL635	This study
mc^{2}5009	IKEPLUS_1			(2)

Table S2: List of plasmids and phasmids used in the current study: Abbreviations used *hyg*^{R}: hygromycin resistance, *suc*^{S}: Sucrose sensitive, *kan*$^{R:}$ Kanamycin resistance.

Plasmids/phages	Description	Reference
pYUB1136	$attP_{L5}\lambda cosColE1$ *apr bla*	(2)
pYUB1336	pYUB1136: :(Rv0278-Rv303)	(2)
pYUB2098	pYUB1336(Rv0278-Rv303)::*kan*^{R}	This study
pYUB2099	Gamma delta Resolvase, *hyg*, *sacB* (to remove integrase)	This study
pBRL635	Integrative plasmid with *leuCD*	Tiwari et al. (unpublished)
phAE159	Conditionally replicating shuttle phasmid vector	(2)
phAE543	phAE159::pYUB1432	(2)

phAE763	ph159 with pYUB1572	Tiwari et al. (unpublished)

Table S3: Primers used in the current study.

Name of Primer	Sequence	Purpose
IKEPLUS_F setI (12907-13966)	GCCTCGACAGTTAGCTTATGCAATG	To confirm complementation
IKEPLUS_R setI (12907-13966)	AACTCGGCGAGTTGGAGTTCG	To confirm complementation
IKEPLUS_F setII (22944-23441)	ACCGCACGACAGCAAGTAAC	To confirm complementation
IKEPLUS_R setII (22944-23441)	CCAAACCGACACCAAGAATCGG	To confirm complementation
Primers for Q-PCR		
16S-RT-F-Mo	GCC GTA AAC GGT GGG TAC TA	Primers for Q-PCR
16S-RT-R-Mo	TGC ATG TCA AAC CCA GGT AA	Primers for Q-PCR
Rv0282-RT-F-Mo	ATT TCC ACC TCG CGT ATG CC	Primers for Q-PCR
Rv0282-RT-R-Mo	TGA GCA GCT TCA CGA CAT CC	Primers for Q-PCR

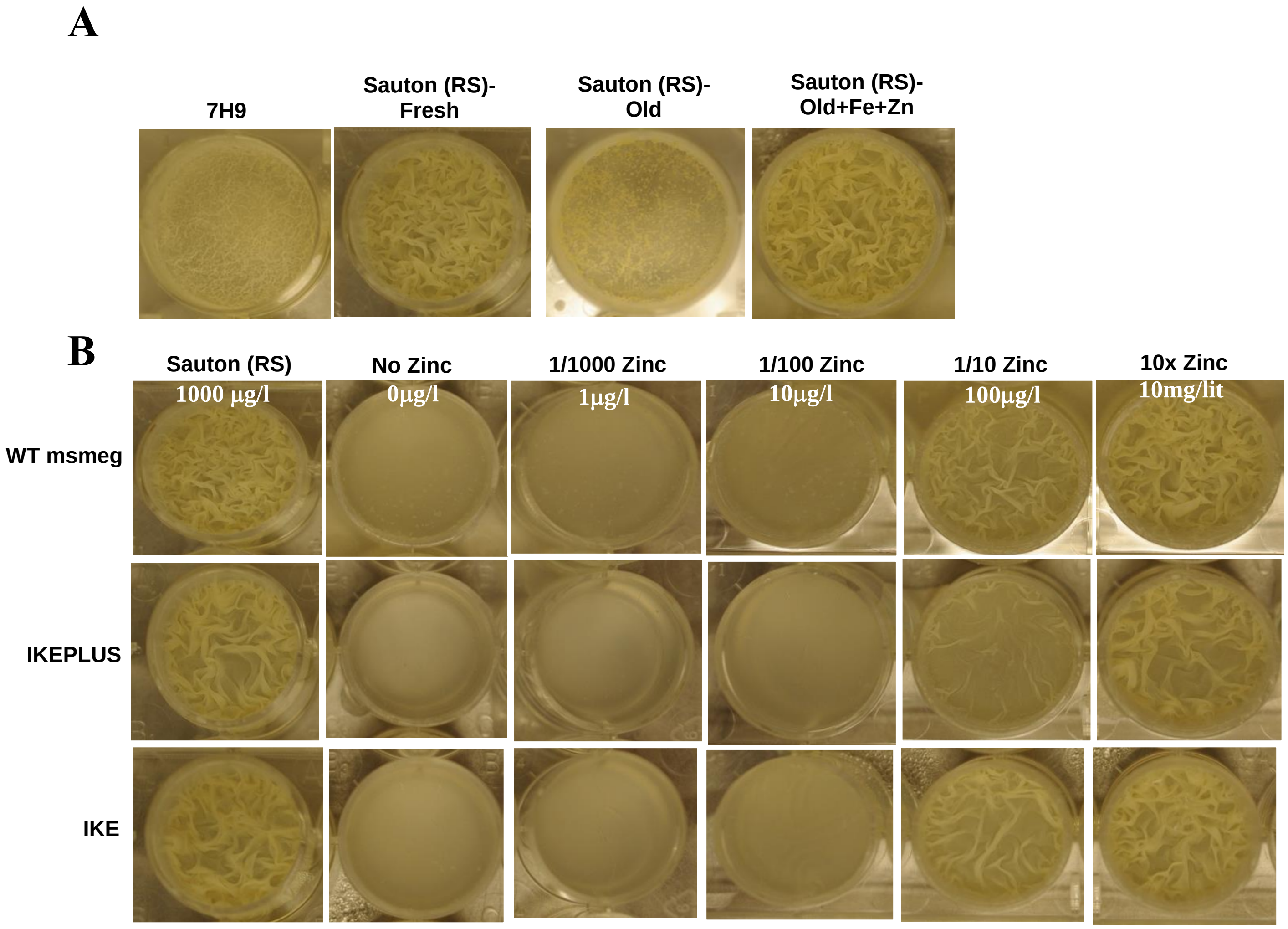

Supplementary Figure 1: Zinc and Iron affect the growth of *M. smegmatis* in Sauton medium. Figure S1A: Biofilm assays showed that wild type *M. smegmatis* mc^{2}155 grown in 7H9 media formed thin biofilms but the same strain grown in Sauton medium formed thick biofilms. Additionally, when old Sauton medium was supplemented with zinc and iron, *M. smegmatis* growth was augmented. **Figure S1B.** Next, we investigated the role of zinc on the growth of wild type *M. smegmatis*, IKEPLUS and IKEPLUS. Zinc concentrations were titrated to support the growth of *M. smegmatis*. We found that zinc plays a vital role in the growth and formation of *M. smegmatis* biofilms, and 100ug/l of zinc sufficiently supported the growth of *M. smegmatis*.

References:

1.Snapper SB, Melton RE, Mustafa S, Kieser T, Jacobs WR, Jr. Isolation and characterization of efficient plasmid transformation mutants of Mycobacterium smegmatis. Mol Microbiol. 1990;4(11):1911-9.

2.Sweeney KA, Dao DN, Goldberg MF, Hsu T, Venkataswamy MM, Henao-Tamayo M, et al. A recombinant Mycobacterium smegmatis induces potent bactericidal immunity against Mycobacterium tuberculosis. Nat Med. 2011;17(10):1261-8.
